# Detecting Alcohol-Related Problems in Trauma Center Patients

**Published:** 1994

**Authors:** Carl A. Soderstrom

**Affiliations:** Carl A. Soderstrom, M.D., is an assistant professor of surgery at the University of Maryland School of Medicine and the director of physician education in the Department of Surgical Traumatology, R Adams Cowley Shock Trauma Center, University of Maryland Medical Center, Baltimore, Maryland

## Abstract

Injury and alcohol are strongly associated. Trauma center clinicians have a unique opportunity to prevent future injuries by identifying patients with alcohol-related problems.

Alcohol-related impairment has been linked to an array of serious and fatal injuries resulting from various mechanisms of injury, including traffic crashes, falls, domestic violence, suicide, and assault. Most victims of trauma are treated in a hospital emergency department (ED) and released. More seriously injured victims, however, may be treated at 1 of the more than 400 trauma centers nationwide. Here, clinicians not only treat physical injuries but also have the opportunity to detect alcohol-related problems^1^ that might otherwise go unrecognized.

As demonstrated by Gentilello and colleagues (1988), alcoholic trauma patients are amenable to entering treatment programs; however, current practices in trauma centers do not facilitate that process. Fewer than 25 percent of trauma centers routinely test for alcohol and other drugs of abuse, obtain patients’ histories of alcohol abuse, and employ full-time drug dependence counselors (Soderstrom and Cowley 1987).

This article discusses the need for alcohol use assessment as a routine part of trauma center care and evaluates some screening tests for usefulness for this purpose.

## Reasons for Screening

Two trauma center studies highlight the need to identify and treat trauma patients who suffer from alcoholism. A Detroit study (Sims et al. 1989) of victims of violence noted that those patients with evidence of alcohol abuse were almost twice as likely (48 percent vs. 28 percent) to sustain a second injury within 5 years that required either ED treatment or readmission to a trauma center than were patients without evidence of alcohol abuse.

A Seattle study (Rivara et al. 1993*a*), with an average followup time of more than 2 years, assessed the relative risks of trauma patients sustaining a second traumatic episode requiring readmission. Among those who were intoxicated at the time of the first admission, those judged possibly alcoholic based on a screening interview, and those who had elevated gamma-glutamyl transferase (GGT) levels (see below), the relative risks of readmission for injury compared with the risks among subjects without those characteristics were 2.5, 2.2, and 3.5 times higher, respectively.

Commenting in part on the problem of alcohol-related repeat trauma episodes, Gordis (1991) noted

Although some progress has been made to improve early identification and referral of alcohol abusers by physicians and other health care personnel, we still have not reached the point where attention to a patient’s alcohol use pattern is a routine part of medical care (p. 3).

## Effect of Alcohol on Recovery From Trauma

Excessive use of alcohol can cause several bodily changes that might increase illness and death rates among trauma patients (Soderstrom 1989; Albin 1987; Gentilello et al. 1993). Alcohol abuse could exacerbate conditions such as brain, spinal cord, and heart injuries; shock resulting from blood loss; impaired clotting of the blood; and infectious diseases that may follow trauma.

Large studies of trauma patients in which outcome was assessed relative to living or dying have suggested that alcohol use immediately prior to trauma either has no effect on survival (Soderstrom and Eastham 1987) or appears to be associated with increased chances of survival (Ward et al. 1982). As stated by Waller (1988), the methodologies that were used to assess alcohol’s effect on mortality in studies such as those cited above may be

flawed because nothing is known about kinetic or other energy forces causing injuries, and analysis may be limited only to inpatients, with no knowledge about those not injured, those with injuries not requiring treatment, those seen only in the emergency department, or those who died at the scene (p. 1632).

In a North Carolina study of more than 1 million vehicular crash victims, Waller and colleagues (1986) found that when age, gender, vehicle weight, and deformation incurred in the crash, and other factors were considered, the intoxicated driver was more likely to die or suffer serious injury. In a Maryland study, Dischinger and colleagues (1988) noted that vehicular crash victims who were intoxicated were more likely to die at the scene of injury than were nonintoxicated crash victims.

A recent Seattle study (Jurkovich et al. 1993) obviated many of the previous trauma center study concerns expressed by Waller (1988). Results indicated that acute intoxication had no effect on the death of trauma patients relative to place of death, that is, either at the scene or within or after 24 hours after admission to the trauma center. The study did demonstrate a higher risk of complications, particularly infections, among patients with evidence of chronic alcohol abuse compared with other patients. In another Seattle trauma center study, Gentilello and colleagues (1993) demonstrated an association between the occurrence of infections in patients who had penetrating wounds and a blood alcohol concentration (BAC) of at least 0.2 percent at the time of admission. An additional Seattle study (Gurney et al. 1992) suggested that intoxicated patients with brain injuries are at greater risk of developing pneumonia and respiratory distress than are nonintoxicated patients.

Overall, there is a dearth of outcome studies of the type mentioned above. Alcohol affects multiple organs and organ systems, and its effects will vary according to age of onset of alcohol use, years of use, drinking patterns, gender, preexisting diseases, and a host of other factors.

## Patient Profile

Each year, approximately 800 to 1,000 people per million sustain injuries requiring treatment in a trauma center (National Highway Traffic Safety Administration 1987). Demographic information for 16,251 patients treated at the R Adams Cowley (RAC) Shock Trauma Center in Baltimore, MD, during a 6-year period from July 1987 to June 1993 is presented in table 1. This free-standing trauma center is the core facility of Maryland’s comprehensive system of emergency trauma care. The gender, age, and mechanism of injury profiles of the center’s patients reflect those of patients treated in trauma centers throughout the United States (Champion et al. 1990).

BAC is determined for more than 90 percent of patients admitted to the RAC Shock Trauma Center from the scene of injury. As shown in table 1, alcohol use immediately prior to injury is highest among men, patients 21 to 49 years of age, motorcyclists, struck pedestrians, and victims of intentional violence. The rates of preinjury alcohol use reported in table 1 are similar to those found at other centers for victims of both intentional and unintentional injury (Rivara et al. 1993*b*; Sloan et al. 1989; Lindenbaum et al. 1989). Alcohol is the drug most frequently detected in trauma patients; however, a spectrum of other drugs is found also, either alone or in combination with alcohol (Sloan et al. 1989; Lindenbaum et al. 1989; Rivara et al. 1989; Soderstrom et al. 1988).

## Assessing Alcoholism in Trauma Center Patients

Almost a decade ago, Reyna and colleagues (1985) stated, “An [ED] visit for loss of consciousness, abdominal trauma, or even seemingly insignificant lacerations and fractures may be the first and only opportunity the surgeon has to identify undercover alcoholics” (p. 197). Taking advantage of that opportunity may be difficult, because the ED is geared for immediate treatment of patients’ injuries, after which most patients are sent directly home. Also, patients with elevated BAC’s are usually in no condition to benefit from counseling services prior to leaving the ED.

On the other hand, admission to a trauma center allows identification, counseling, and referral for treatment of an addiction problem. However, a national survey, documenting responses from 316 trauma centers, revealed that although resources to perform BAC testing were available at all but 2 of those centers (99.4 percent), BAC testing was a standard clinical practice at only one-third (38.3 percent) (Soderstrom et al. 1994). The predominant reason given for not obtaining BAC’s is that the test is regarded as “clinically not important.” These results did not vary significantly from a survey conducted 5 years earlier (Soderstrom and Cowley 1987). In addition, fewer than 40 percent of the responding centers did not test routinely for other drugs (Soderstrom et al. 1994).

The “clinically not important” response of trauma center clinicians regarding alcohol testing probably reflects a general disinterest in alcoholism and other chemical dependence problems. A study of Johns Hopkins medical students and residents by Geller and colleagues (1989) suggests that those who were “further along” in their education were more likely to regard alcoholism as a “character weakness” and to believe that treatment/rehabilitation “does not work” (pp. 3117–3118).

Similarly, Chang and colleagues (1992) found in a survey of more than 1,000 emergency medicine specialists that most considered alcoholics “difficult to treat.” These specialists did agree that alcoholism is a “treatable disease” but indicated that neither physicians (excluding psychiatrists) nor surgeons are effective clinicians for the treatment of alcoholism. These attitudes are not surprising: because the treatment of life-threatening conditions must take precedence in ED’s and trauma centers, the identification and treatment of alcoholism is deemed a secondary concern.

## Indicators of Alcoholism in Trauma Center Patients

Several indicators can be used to help clinicians screen for alcohol problems among patients in trauma centers. Potential indicators include intoxication on admission, presence of cirrhosis, levels of liver enzymes and other biological markers, results of interview questionnaires, and a history of other drug use. No single test is sufficient for screening this population. Information from demographic data must be linked to physiologic data, BAC and other toxicology results, and interview results to identify those trauma patients who require a formal evaluation by addiction clinicians.

### Intoxication

Many injured drivers admitted to trauma centers are intoxicated at the time of admission (Rivara et al. 1993*b*; Soderstrom et al. 1988; Stoduto et al. 1993). Intoxication should be considered a strong indicator of possible alcoholism. For example, psychiatric evaluations of drivers convicted of alcohol-impaired driving indicated that about one-third had a diagnosis of alcohol dependence and about one-half could be characterized as having an alcohol abuse disorder (Kruzich et al. 1986; Miller et al. 1986).

### Cirrhosis

The result of a prolonged history of heavy alcohol consumption, cirrhosis can be detected through laboratory testing of liver function. However, biochemical evidence of liver dysfunction is not detected as frequently as expected among trauma victims, probably because this population tends to be younger than the classic alcoholic with liver disease. (The average patient with alcoholic cirrhosis has been drinking heavily for 10 to 20 years [Grant et al. 1988].)

In a study of preexisting disease among 27,029 trauma patients discharged alive from California hospitals, a discharge diagnosis of cirrhosis was recorded for only 0.5 percent. The highest rates were in the 45- to 54-year and the 55- to 64-year age groups, being 1.1 percent and 1.5 percent, respectively (MacKenzie et al. 1989).

In a review of trauma registry data from two Pennsylvania trauma centers involving more than 27,000 patients over a 9-year period, Tinkoff and colleagues (1990) found only 40 (1.4 percent) patients with cirrhosis. The mean age of the patients in that diagnostic group was 58 years. In another trauma registry review of almost 8,000 patients admitted to the RAC Shock Trauma Center, mentions of cirrhosis were noted less than 1 percent of the time (Milzman et al. 1992). These studies suggest that cirrhosis is rarely encountered in trauma centers. Hence, a search for that condition will provide a low yield for identifying trauma patients with alcoholism.

### Biological Markers

In a recent study, Rivara and colleagues (1993*b*) assessed levels of GGT and glutamate dehydrogenase (GDH) among both intoxicated (BAC at least 0.1 percent) and nonintoxicated patients admitted to Seattle’s Harborview Medical Center. GGT and GDH are liver enzymes whose levels in the blood are used as markers of liver disease (for more information on biological markers, see the article by Salaspuro, pp. 131–135). Overall, 47.0 percent of the 2,657 patients had an elevated BAC, of whom 76 percent were intoxicated. Sixty percent of the intoxicated patients were younger than 35 years of age. Among the nonintoxicated patients, 11.4 percent had elevated GGT levels and 27.6 percent had elevated GDH levels, compared respectively with 28.0 percent and 35.6 percent of the intoxicated patients. In both intoxicated and nonintoxicated patients, the highest percentages of abnormal enzyme elevations were found in age groups above 45 years.

In a study assessing the value of liver function tests to predict injury to the liver and other abdominal organs, Sahdev and colleagues (1991) measured the liver enzymes glutamic oxaloacetic transaminase (SGOT) and glutamic pyruvic transaminase (SGPT) in the serum (liquid part of the blood) of 309 patients. Levels of these enzymes were normal in 80.9 percent of the patients. However, the researchers found a significant association between elevated SGOT or SGPT levels and alcohol levels.

Test results for currently available markers do not yield a high rate of specificity^2^ in the diagnosis of alcoholism. For example, in one Seattle study (Rivara et al. 1993*b*), only 30.4 percent of the intoxicated patients with results suggestive of alcoholism, based on a frequently used test, had elevated GGT levels. GDH also appeared to be a poor marker for alcoholism in trauma patients. Measurement of an abnormal blood protein called carbohydrate-deficient transferrin (CDT) may prove to be a more accurate method of detecting recent alcohol abuse (Stibler 1991). The CDT marker test, which has not been approved for diagnostic clinical use, has yet to be studied in a population of trauma patients.

### Patient Interviews

In studies at the RAC Shock Trauma Center (Soderstrom et al. 1992) and Seattle’s Harborview Medical Center (Rivara et al. 1993*b*), BAC test results of intoxicated and nonintoxicated patients were compared with interview results. In both studies, interview results led to a diagnosis of alcoholism in significant numbers of patients who had no detectable BAC at the time of admission.

In the RAC Shock Trauma Center study, 11 of 24 (46 percent) patients with BAC’s of 0 had a history of alcohol dependence at some time in their lives. In the Seattle study (Rivara et al. 1993*b*), 394 of 1,540 (26 percent) nonintoxicated patients (BAC = 0 or less than 0.1 percent) had interview results indicative of possible alcoholism. These observations suggest that the search for alcoholics in trauma patients should not be limited to those with elevated BAC’s.

## Summary

Injury and alcohol are strongly associated. Patients admitted to trauma centers are frequently intoxicated. Many of those patients require identification and treatment of an underlying drug use problem, most commonly alcoholism. Admission to the trauma center provides clinicians with an opportunity to accomplish that task, which should lead to significant reduction in illness and death secondary to injury. To date, the primary emphasis of trauma center care for alcoholic patients has focused on the treatment of physical injuries. The potential to prevent future injury by identifying and treating alcoholism and other drug problems, however, has been largely ignored.

## Figures and Tables

**Table 1:** Blood Alcohol Concentration (BAC) of Patients Admitted to the R Adams Cowley Shock Trauma Center, July 1987–June 1993

	Number	BAC-Positive On Admission (percent)	Mean BAC (percent)
Gender			
Men	11,734	36	0.159
Women	4,517	20	0.155
Age (years)			
≤ 20	2,934	21	0.116
21–35	7,611	40	0.158
36–49	3,102	33	0.178
≥ 50	2,604	17	0.175
Type of Injury			
Unintentional	12,780	29	0.158
Vehicular			
Automobile^1^	7,967	31	0.153
Motorcycle^2^	962	42	0.141
Pedestrian	1,072	41	0.189
Other^3^	2,779	16	0.168
Intentional^4^	3,471	40	0.162

1 Vehicular occupant: driver or passenger.

2 Vehicular occupant: driver or passenger.

3 Falls, work-related and recreational injuries, etc.

4 Assaults, gunshot and knife wounds, etc.

SOURCE: National Study Center for Trauma and Emergency Medical Systems, University of Maryland 1994.

## Footnotes

1 Throughout this article, terms such as “alcohol abuse,” “alcoholism,” and “heavy drinking” are used. There is overlap among these terms in the alcoholism literature; therefore, the wording in each case is based on the terms used in the reference cited.

2 Specificity represents a test’s ability to rule out disease in a patient who does not have the disease.
